# Beetroot Juice Supplementation Improves High-Intensity Intermittent Type Exercise Performance in Trained Soccer Players

**DOI:** 10.3390/nu9030314

**Published:** 2017-03-22

**Authors:** Jean Nyakayiru, Kristin L. Jonvik, Jorn Trommelen, Philippe J. M. Pinckaers, Joan M. Senden, Luc J. C. van Loon, Lex B. Verdijk

**Affiliations:** 1 Department of Human Movement Sciences, NUTRIM School of Nutrition and Translational Research in Metabolism, Maastricht University Medical Centre+, P.O. Box 616, 6200 MD Maastricht, The Netherlands; jean.nyakayiru@maastrichtuniversity.nl (J.N.); kristin.jonvik@maastrichtuniversity.nl (K.L.J.); jorn.trommelen@maastrichtuniversity.nl (J.T.); philippe.pinckaers@maastrichtuniversity.nl (P.J.M.P.); joan.senden@maastrichtuniversity.nl (J.M.S.); l.vanloon@maastrichtuniversity.nl (L.J.C.v.L.); 2 Institute of Sports and Exercise Studies, HAN University of Applied Sciences, P.O. Box 6960, NL 6503 GL Nijmegen, The Netherlands

**Keywords:** football, nitrate, nitrite, nitric oxide, ergogenic aid

## Abstract

It has been shown that nitrate supplementation can enhance endurance exercise performance. Recent work suggests that nitrate ingestion can also increase intermittent type exercise performance in recreational athletes. We hypothesized that six days of nitrate supplementation can improve high-intensity intermittent type exercise performance in trained soccer players. Thirty-two male soccer players (age: 23 ± 1 years, height: 181 ± 1 m, weight: 77 ± 1 kg, playing experience: 15.2 ± 0.5 years, playing in the first team of a 2nd or 3rd Dutch amateur league club) participated in this randomized, double-blind cross-over study. All subjects participated in two test days in which high-intensity intermittent running performance was assessed using the Yo-Yo IR1 test. Subjects ingested nitrate-rich (140 mL; ~800 mg nitrate/day; BR) or a nitrate-depleted beetroot juice (PLA) for six subsequent days, with at least eight days of wash-out between trials. The distance covered during the Yo-Yo IR1 was the primary outcome measure, while heart rate (HR) was measured continuously throughout the test, and a single blood and saliva sample were collected just prior to the test. Six days of BR ingestion increased plasma and salivary nitrate and nitrite concentrations in comparison to PLA (*p* < 0.001), and enhanced Yo-Yo IR1 test performance by 3.4 ± 1.3% (from 1574 ± 47 to 1623 ± 48 m; *p* = 0.027). Mean HR was lower in the BR (172 ± 2) vs. PLA trial (175 ± 2; *p* = 0.014). Six days of BR ingestion effectively improves high-intensity intermittent type exercise performance in trained soccer players.

## 1. Introduction

While nitrate and nitrite were previously considered inert byproducts of the nitric oxide (NO) metabolism, recent insights suggest that (dietary) nitrate can also serve as a precursor for NO through the nitrate -> nitrite -> NO-pathway [1]. Different studies have shown that both plasma nitrate and nitrite concentrations increase following dietary nitrate supplementation in a dose-dependent manner [2,3]. These elevations in plasma concentrations have in turn been associated with improvements in exercise performance, suggesting ergogenic benefits from activation of the nitrate to NO pathway [4,5,6].

Multiple studies from different laboratories have shown that dietary nitrate ingestion can decrease the oxygen cost of submaximal exercise and increase high-intensity exercise tolerance in recreational athletes [4,7,8]. Furthermore, we have previously shown that nitrate-rich beetroot juice ingestion can not only increase oxygen efficiency during submaximal cycling exercise, but that it can also improve time trial performance in moderately trained cyclists and triathletes [5]. As such, this work, in line with others [6,9], has established a functional benefit of dietary nitrate supplementation on exercise performance.

Most of the earlier work on the ergogenic effects of nitrate supplementation was focused on endurance type sports, while little attention has been given to high-intensity and/or intermittent type exercise performance. However, recent findings suggest that nitrate might largely convey its effects on exercise performance through type II muscle fibers [10,11]. Ferguson et al. [10] used a rat model to assess the effects of dietary nitrate supplementation on blood flow in vivo during submaximal exercise. The increases in blood flow and vascular conductance in the exercising limbs were primarily observed in fast twitch muscle fibers. In line with these observations, Hernandez et al. [11] reported that dietary nitrate supplementation improves intracellular calcium handling in fast-twitch muscles of mice, which resulted in increased force production. Based on these findings in rodents, it could be suggested that the ergogenic effects of nitrate might be most profound for activities that recruit type II muscle fibers [10,11], i.e., (very) high-intensity exercise bouts of short duration.

Soccer is one of the world’s most widely performed team sports and is characterized by players performing multiple bouts of high-intensity running and sprinting throughout the 90 min of a match, during which there is heavy reliance on the contribution of type II muscle fibers [12]. These periods of high-intensity activity are alternated with periods of relative recovery, resulting in an intermittent type intensity profile [12,13,14]. The Yo-Yo Intermittent recovery test level 1 (Yo-Yo IR1) is an often used measurement tool to simulate these soccer specific activities in a controlled setting, thereby allowing the reliable and feasible assessment of physical performance in soccer players [15]. Indeed, the Yo-Yo IR1 test has been shown to cover aspects of both aerobic as well as anaerobic performance in soccer players, with a strong link towards the ability to perform high-intensity intermittent type exercise throughout a match [12,15]. Using the Yo-Yo IR1, two previous studies described improved high-intensity intermittent type exercise performance following nitrate-rich beetroot juice ingestion in recreationally active team sport players [16,17]. These observations were the first evidence of ergogenic benefits that team sport players (such as soccer players) could have from nitrate ingestion. The earlier of the two studies observed these effects after ingestion of a high nitrate dose (1780 mg, 28.7 mmol) in the 30 h prior to the high-intensity intermittent running test [16]. Although effective, the dosing strategy that was applied in the study strongly deviates from that of current multiday supplementation protocols that have proven effective in endurance athletes [4,5,7]. More in line with current nitrate supplementation regimens, Thompson et al. recently concluded that a five-day nitrate supplementation protocol with a lower daily dose of nitrate was also effective in improving high-intensity intermittent running performance in recreational athletes [17]. Extending on this finding in recreational athletes, we hypothesized that a homogenous group of trained soccer players performing intermittent type exercise would also benefit from nitrate ingestion. Therefore, we assessed the effects of a six-day nitrate-rich beetroot juice supplementation protocol on high-intensity intermittent running performance in a group of trained soccer players.

## 2. Materials and Methods

### 2.1. Subjects

A total of 40, first team, male soccer players competing in the 2nd and 3rd Dutch amateur league were recruited to participate in the study. After being informed about the purpose and potential risks of the study, all subjects provided written informed consent. The experimental protocol and procedures were approved by the medical ethical committee of the Maastricht University Medical Centre, the Netherlands (METC 153006; ClinicalTrials.gov: NCT02436629). Eight subjects failed to complete the study because of injury (*n* = 3), failure to comply with the protocol (dietary/activity standardization procedures; *n* = 4), or due to personal time constraints (*n* = 1). Data of the remaining 32 subjects (age: 23 ± 1 years, height: 181 ± 1 cm, weight: 77 ± 1 kg, BMI: 23.4 ± 0.4 kg/m^2^, playing experience: 15.2 ± 0.5 y) was used in the analysis. 

### 2.2. Study Design

This double blind, randomized, placebo-controlled, cross-over study was designed to investigate whether six days of nitrate-rich beetroot juice (BR) supplementation improves intermittent type exercise performance in trained soccer players. Subjects were required to report to the research facility on four occasions, spread over a three-week period. Following a screening session (visit one), subjects visited the research facility ~1 week prior to the first experimental trial to get familiarized with the Yo-Yo intermittent Recovery test level 1 (Yo-Yo IR1) and to receive their supplemental beverages (visit two). No blood or saliva samples were collected during familiarization. The experimental trial days (visits three and four) that followed were each on day six of the nitrate-rich or nitrate-depleted beetroot juice supplementation period, with the last supplemental bolus being ingested 3 h prior to performing the Yo-Yo IR1. Wash-out between the two supplementation periods was at least eight days.

### 2.3. Supplementation Protocol and Standardization of Physical Activity and Diet

During the two 6-day supplementation periods, subjects ingested 2 × 70 mL/day of beetroot juice. The choice for beetroot juice was largely based on previous observations by us [18], and by others [19], that suggest more pronounced benefits from nitrate ingestion through plant based sources than following sodium nitrate ingestion. The daily 140 mL bolus of nitrate-rich beetroot juice (BR) provided ~800 mg of nitrate (~12.9 mmol), while the beetroot juice placebo (PLA) was similar in taste and appearance but instead was depleted of nitrate (both supplied by Beet It, James White Drinks Ltd., Ipswich, UK). Subjects were instructed to ingest the 2 × 70 mL shots around the same time each day (~5 pm), which was based on the time the final bolus was ingested on day six of supplementation; i.e., 3 h prior to the exercise test. In addition, subjects recorded their activities and dietary intake in the 36 h prior to the first experimental trial, which were then replicated in the 36 h prior to the second trial. Subjects refrained from strenuous physical exercise or labor in the 48 h leading up to the trial days, and did not consume caffeine or alcohol in the 12 h and 24 h prior to each trial, respectively. To prevent any attenuation in the reduction of nitrate to nitrite by commensal bacteria in the oral cavity, subjects refrained from using antibacterial mouthwash/toothpaste and chewing gum during the six-day supplementation periods [20]. No restriction was set for the consumption of nitrate-rich foods. This was done to allow for the determination of the additional effect of dietary nitrate on performance, on top of the normal diet. As has also been done previously [21], on test days, all subjects were provided with a standardized dinner that was consumed ~3.5 h prior to the exercise test. After consumption of this meal and the final supplemental bolus, subjects were only allowed to consume an *ad libitum* amount of water in the hours that followed. The amount of water consumed before and during the first trial was replicated during the second trial.

### 2.4. Experimental Protocol

On the last day of each supplementation period, subjects reported to the research facility ~2 h after ingesting the last 140 mL bolus of beetroot juice. The trials started with collection of a single antecubital venous blood sample and collection of a saliva sample for determination of pre-exercise nitrate and nitrite concentrations (2.5 h after ingesting the last supplemental bolus). Subjects then filled out a gastrointestinal (GI) tolerance questionnaire to assess GI complaints as a result of supplement ingestion. A heart rate monitor (Zephyr Technology Corporation, Annapolis, MD, USA) was then fitted before subjects performed a standardized 10-min warm-up, after which the Yo-Yo IR1 was performed. Heart rate was monitored continuously (1 Hz) to calculate mean heart rate throughout the test, as well as peak heart rate reached near the end of the Yo-Yo IR1 (30-s peak heart rate).

The warm-up and the Yo-Yo IR1 were performed indoors in a sports hall, on a 2 by 20 m running lane that was marked by cones, as described previously by Krustrup et al. [15]. The test consisted of repeated 2 × 20 m sprints between a starting, turning, and finishing line at a progressively increasing speed controlled by audio bleeps from an audio system. Between each 2 × 20 min run, subjects had a 10-s active recovery period in an area of 5 × 2 m that was marked by cones behind the start/finishing line. When a subject failed to cross the finish line before the final bleep, a warning was given. When a subject failed to cross the finish line before the bleep for a second time, the final distance covered was registered and represented the end result [15]. Immediately after completing the Yo-Yo IR1, subjects rated their perceived exertion on a Borg 6–20 scale [22].

### 2.5. Plasma and Saliva Analysis

Blood samples were collected in Lithium-Heparin containing tubes and immediately centrifuged at 1000× *g* for 5 min, at 4 °C. Aliquots of plasma were frozen in dry-ice after centrifugation, and were stored at −80 °C for subsequent analysis of plasma nitrate and nitrite concentrations. Saliva samples were collected in 2 mL Eppendorf cups and stored at −80 °C until nitrate and nitrite concentrations were determined in both saliva and plasma using chemiluminescence, as described previously [18].

### 2.6. Statistical Analysis

A sample size of 40 subjects, including a 20% dropout, was calculated with a power of 80% and an alpha of 0.05 (two-sided) to detect a 4.2% difference in the distance covered during the Yo-Yo IR1 between BR and PLA. Performance data from the Yo-Yo IR1, heart rate, and plasma and saliva data were analyzed with a paired samples t-test (BR vs. PLA). Effect size of Yo-Yo IR1 performance was determined using Cohen’s *d*_z_ statistical calculation for paired samples. Heart rate data of 7 subjects was incomplete (due to technical problems and/or shifting of the chest bands) and was therefore not included in the analysis. Pearson correlation coefficients were calculated to assess whether differences in plasma or saliva nitrate and nitrite concentrations between trials were associated with the difference in Yo-Yo IR1 performance or heart rate variables between BR and PLA. Statistical significance was set at *p* < 0.05, and all data were analyzed using SPSS 21.0 (version 21.0, IBM Corp., Armonk, NY, USA), and are presented as means ± SEM.

## 3. Results

### 3.1. Plasma and Saliva Nitrate and Nitrite Concentrations

**Figure 1:**
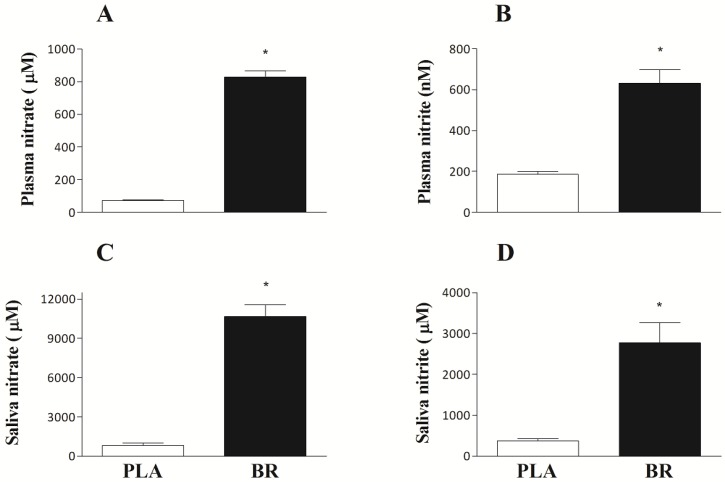
Mean plasma nitrate (**A**) and nitrite (**B**), and saliva nitrate (**C**) and nitrite (**D**) concentrations ~2.5 h after ingestion of the final supplemental bolus for the placebo (PLA) and the six-day nitrate-rich beetroot juice (BR) intervention. Data are means ± SEM (*n* = 32). * BR significantly different from PLA (*p* < 0.001).

Ingestion of BR for six subsequent days resulted in elevated nitrate concentrations when compared to PLA, in both plasma (Figure 1A) and saliva (Figure 1C) (both *p* < 0.001). Similarly, nitrite concentrations were higher following BR vs. PLA supplementation in both plasma (632 ± 66 nM vs. 186 ± 13 nM; *p* < 0.001; Figure 1B) and saliva (2882 ± 519 μM vs. 375 ± 54 μM; *p* < 0.001; Figure 1D).

### 3.2. Yo-Yo IR1 Test

**Figure 2:**
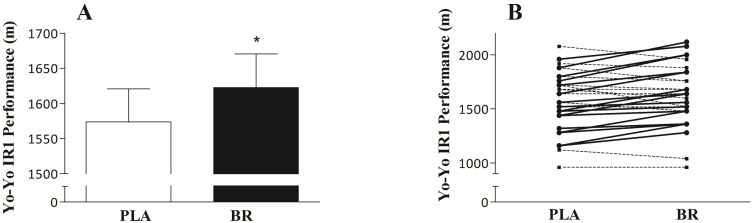
Mean distance covered during the Yo-Yo IR 1 test (**A**), and the individual response (**B**) following 6 days of placebo (PLA) and 6 days of nitrate-rich beetroot juice (BR) ingestion. * Distance covered following BR was significantly greater (3.4%) than that covered following PLA ingestion (*p* = 0.027). Solid lines (-) indicate subjects that showed an improved performance following BR ingestion (*n* = 18). Dashed lines (--) indicate subjects that showed a similar performance (*n* = 4) following BR or PLA ingestion, or subjects that showed a worse (*n* = 10) performance following BR ingestion.

High-intensity intermittent running performance as assessed by the Yo-Yo IR1 significantly improved following BR ingestion (1623 ± 48 m) when compared to PLA (1574 ± 47 m; *p* = 0.027; Figure 2A). The average improvement in distance covered during the test was 3.4 ± 1.3%, with a Cohen’s *d*_z_ of 0.41. Of the 32 subjects assessed, 18 showed an improved performance during the BR trial vs. the PLA trial (+9 ± 5%), 10 had a slightly worse performance (−5 ± 3%) and 4 showed no difference between trials. Although peak heart rate did not differ between trials (*p* = 0.16; Table 1), average heart rate during the Yo-Yo IR 1 test was lower in the BR trial when compared to PLA (*p* = 0.014; Table 1).

### 3.3. GI and Borg Score

Subjects tolerated the interventional drinks well and GI discomfort did not differ between interventions. Only two participants reported a bloated stomach during the PLA trial, and one during the BR trial, while flatulence was reported by two participants during the PLA and two participants during the BR trial. Ratings of perceived exertion as determined with the Borg scale were also not different between interventions (*p* = 0.23; Table 1).

### 3.4. Correlation Analyses

Despite the substantial elevations in plasma and saliva concentrations following BR ingestion, no significant correlations were found between plasma and saliva nitrate (*r* = 0.076, *p* = 0.697) or plasma and saliva nitrite (*r* = 0.264, *p =* 0.144) concentrations. In addition, no associations were observed between (differences in) plasma or saliva concentrations on the one hand, and the (differences in) distance covered, or heart rate variables on the other hand (all *r* ≤ 0.296; all *p* ≥ 0.092).

## 4. Discussion

The current study demonstrates that six days of nitrate-rich beetroot juice supplementation improves high-intensity intermittent type exercise performance in trained soccer players. The improvements in intermittent type exercise performance were accompanied by a lower mean heart rate during the high-intensity intermittent running test, and were preceded by increases in both plasma and saliva nitrate and nitrite concentrations.

Nitrate related research in the past years has primarily focused on establishing the effects of nitrate supplementation on endurance type exercise performance. While improvements in exercise capacity [4,8,23] and exercise performance [5,6] have indeed been observed in endurance athletes, recent literature suggests possible performance benefits of nitrate ingestion in more high-intensity and intermittent type sports and activities [16,24]. Extending on previous observations in recreationally active team-sport players [17], the present study specifically assessed the effects of a multiday supplementation protocol with nitrate-rich beetroot juice on high-intensity intermittent type exercise performance in a large sample of trained soccer players.

We found that six days of BR supplementation elevated nitrate and nitrite concentrations in both plasma and saliva (Figure 1). The observed 11-fold increase in plasma nitrate and 3-fold increase in plasma nitrite concentrations are in line with previous observations where a similar nitrate dose was administered [18,24]. In addition to the changes in plasma concentrations, the current findings suggest that saliva samples might represent a (less invasive) alternative to assess the postprandial response to beetroot juice ingestion. Salivary nitrate and nitrite concentrations were respectively 13-fold and 7-fold higher following BR ingestion when compared to PLA (Figure 1C,D). However, no correlations were observed between plasma concentrations and saliva concentrations. As such, it seems that saliva samples may be used as a means to assess compliance to nitrate supplementation and to confirm the endogenous reduction of nitrate into nitrite. Nevertheless, analysis of salivary nitrate and nitrite does not seem to represent a valid surrogate for quantitative changes in plasma nitrate or nitrite concentrations.

In addition to changes in nitrate and nitrite concentrations, the six-day BR supplementation protocol also resulted in quantifiable improvements in high-intensity intermittent running performance in the soccer players. We observed a 3.4% increase in intermittent type exercise performance on the Yo-Yo IR1 test (Cohen’s *d*_z_: 0.41; Figure 2A). This is in line with a previous report of improvements in high speed running performance in recreationally active team sport players following a multiday BR supplementation regimen [17]. Although the exact mode of action explaining this effect is still unclear, animal studies have shown that nitrate supplementation can increase blood flow [10], and enhance contractile function in type II muscle fibers [11]. There is some suggestion that these adaptations might be responsible for the improved performance observed during high intensity/intermittent type exercise in which type II fibers are heavily recruited [25]. Interestingly, while such cellular changes have been proposed to only occur following a multiday supplementation regimen [10,11,26], two studies from the same laboratory observed improvements in high-intensity intermittent type exercise performance following both an acute high dose BR supplementation protocol (~29 mmol within 36 h; 4.2% improvement) [16], as well as following a five-day BR supplementation approach with a lower daily dose of nitrate (6.4 mmol/day; 3.9% improvement) [17]. The use of a multiday protocol would seem preferred as it likely allows sufficient time for (some of) the suggested cellular adaptations to occur, that might drive the ergogenic effects of nitrate [10,11]. Furthermore, it is believed that trained subjects may require a different nitrate supplementation strategy (i.e., higher dose and/or for a longer period) to elicit beneficial performance effects in comparison to recreational athletes [9,27,28]. The current study therefore assessed the ergogenic effect of a conventional six-day supplementation protocol with BR (12.9 mmol/day nitrate) in a homogenous sample of trained soccer players. Performance on the Yo-Yo IR1 test was on average ~15% higher when compared to the recreational subjects included in the recent study from Thompson et al. [17]. Nonetheless, we observed a 3.4% improvement in high-intensity intermittent running performance, suggesting that a six-day BR supplementation protocol represents a practical and effective regimen for trained soccer players to improve their performance. Clearly, such a performance benefit should be attained without any major negative side effects. In line with previous work [18], only very mild GI discomfort was reported in a few subjects during the current study, supporting the non-adverse use of beetroot juice in relative short-term interventions. Furthermore, as recently reviewed by Bryan and Ivy [29], there is currently no clear indication of adverse health risks accompanying high nitrate intakes for a prolonged period of time. At present, though any potential risks always need to be carefully considered, the established benefits of nitrate, which may be even more pronounced when consuming nitrate through ‘natural’ nitrate-rich vegetable sources [18,19], seem to outweigh the potential risks [29].

Intriguingly, and in contrast to previous studies in team sport players, we observed that ingestion of BR for six consecutive days also had an effect on heart rate during the high-intensity intermittent running test (Table 1). While no changes were observed in peak heart rate, mean heart rate during the Yo-Yo IR1 was lower following BR ingestion than following PLA ingestion. To the best of our knowledge, the current findings are the first evidence of changes in heart rate following nitrate ingestion in young healthy athletes. Whether the decrease in mean heart rate is related to the improved exercise performance is unclear, as the only available literature describing effects of inorganic nitrate-nitrite on heart rate are from heart failure patients [30,31]. Borlaug and colleagues showed that a nitrite infusion protocol in heart failure patients increased cardiac output during exercise [30]. The observed increase in stroke volume was suggested to be explained by improved contractility of the left ventricle. While it is currently unclear whether nitrate and/or nitrite ingestion can similarly increase cardiac contractility in healthy individuals, such an effect could explain the decrease in heart rate observed in our study; i.e., allowing the same cardiac output with increased stroke volume, but lower heart rate. Interestingly, a recent study in rodents also showed increased cardiac contractility following nitrate ingestion, most likely as a result of enhanced expression of calcium handling proteins [32]. As nitrate ingestion has also been shown to enhance expression of calcium handling proteins in type II skeletal muscle fibers [11], such an explanation would fit with both the observed increase in intermittent type exercise performance, and the lower mean heart rate in the current study.

Although nitrate supplementation increased plasma and saliva nitrate and nitrite concentrations, improved exercise performance, and reduced heart rate, no correlations were observed between any of these parameters. Only a limited number of studies have been able to show correlations between plasma concentrations and subsequent performance benefits [2,17,24,27]. In the present study, only a single sample of saliva and plasma was collected ~30 min prior to the exercise test. It could be suggested that a time point closer to, or even during the exercise test may have revealed a relation between plasma concentrations and changes in performance. Despite the fact that all subjects showed substantially increased plasma and saliva nitrate and nitrite concentrations, not all subjects showed improvements in performance (Figure 2B). It is unclear what the exact explanation is for this lack of effect, although it seems likely that the large day-to-day variability inherent to the Yo-Yo test played a role [33] (Figure 2B). Taking this variability into account, the inclusion of a large sample of trained soccer players allowed us to show a significant and relevant improvement in Yo-Yo IR1 test performance following BR ingestion. Importantly, Yo-Yo IR1 performance has been described to strongly correlate with the ability to perform high speed running and sprinting activities throughout a soccer match [15]. As such, our findings suggest that nitrate supplementation could represent an effective nutritional strategy to improve exercise performance in soccer players, especially towards the end of the match when sprint intensity/frequency has been shown to decrease significantly due to fatigue [34]. Even though in general, day-to-day variation in exercise performance tests combined with small sample sizes make it difficult to study potential ergogenic benefits in highly-trained athletes, future work should be undertaken to establish whether these performance improvements in high-intensity intermittent-type exercise in trained soccer players can also be translated toward the elite level.

## 5. Conclusions

Based on the present findings in a large sample of trained soccer players, we conclude that six days of nitrate-rich beetroot juice ingestion improves high-intensity intermittent type exercise performance.

## Figures and Tables

**Table 1:** Heart rate data and Rate of perceived exertion.

Variable	PLA	BR
Mean heart rate (bpm)	175 ± 2	172 ± 2 *
30-sec max heart rate (bpm)	191 ± 1	190 ± 1
RPE (Borg score)	17.6 ± 0.3	17.3 ± 0.4

All values are means ± SEM (*n* = 25 for HR and *n* = 32 for RPE). * Significantly different from PLA (*p* < 0.05).
